# Influence of Tourism Economy on Air Quality—An Empirical Analysis Based on Panel Data of 102 Cities in China

**DOI:** 10.3390/ijerph19074393

**Published:** 2022-04-06

**Authors:** Fen Zhang, Haochen Peng, Xiaofan Sun, Tianyi Song

**Affiliations:** 1 School of Economics and Management, Wuhan University, Wuhan 430072, China; penghaochen@whu.edu.cn; 2 School of Foreign Studies, Xi’an Jiaotong University, Xi’an 710049, China; sxf1245888577@stu.xjtu.edu.cn; 3 College of Business, City University of Hong Kong, Hong Kong 999077, China; tianysong4-c@my.cityu.edu.hk

**Keywords:** tourism economy, air pollution, environmental Kuznets curve, penal VAR, Geodetector

## Abstract

The relationship between regional tourism development and air quality is complex. Although air pollution restricts tourists’ willingness to travel, the air pollution produced by tourism and its ancillary industries can also not be ignored. Using the annual panel data of PM_2.5_ concentration and tourism revenue at the city level, and comprehensively using the Panel VAR model, Geodetector and other analysis methods, we explored the spatio-temporal relationship between the tourism economy and its impact on air quality in China. The main conclusions are as follows: first, the “Kuznets” curve of tourism development and air pollution in mainland China from 2004 to 2016 is generally significant—that is, the tourism economy and air pollution generally show an “inverted U-shaped” relationship. Second, the tourism economy has a positive effect on air pollution in the short term, and this effect is stronger in the eastern region. Third, tourism economy is not the leading factor affecting the change in regional air pollution. GDP and industrial structure are more likely to have the greatest impact on air pollution, and the effect of this “joint force” factor on air pollution is greater than that of other single factors. In the future, the high-quality development of China’s tourism economy needs to take environmental protection into consideration, and advocate for low-carbon travel and green tourism.

## 1. Introduction

In the modern era, the development of tourism plays a very important role in promoting the transformation and upgrading of the regional economy. According to statistics from the Ministry of Culture and Tourism of China, in 2018, the number of domestic tourists in mainland China was 5.539 billion, the total number of inbound and outbound tourists was 291 million, and the total tourism revenue was 5.97 trillion yuan. In 2018, the comprehensive contribution of tourism to GDP was 9.94 trillion yuan, accounting for 11.04% of the total GDP, which was equivalent to the GDP of Guangdong Province in that year; the number of direct and indirect employees in tourism was 79.91 million, accounting for 10.29% of the total employed population in China. In the same yearalthough the air quality had improved to a certain extent, cities reaching air quality standards only accounted for 35.8% of total cities in China. Although tourism is a less polluting tertiary industry, its impact on the environment cannot be ignored. On the one hand, the development of tourism can improve the ecological environment. For example, the vigorous development of ecotourism has improved the quality of the environment. On the other hand, some tourism activities such as transportation will inevitably have a negative impact on the ecological environment. In addition, environmental pollution will also affect tourism motivation and behaviors, which in turn restrict the development of tourism.

The relationship between tourism development and the ecological environment has always been a hotspot of academic research. It is generally believed that environmental pollution mainly includes air pollution, water pollution and solid waste pollution. However, tourists have the strongest perception of air pollution when they travel abroad. Does air pollution drive away tourists and thus affect the growth of the tourism economy? Will the rapid development of tourism and its ancillary industries have any effects on the environment? The international literature has paid much attention to this problem previously, and this issue has been summarized with the the “environmental Kuznets curve (*EKC*)” [1]. Some scholars support this theory [2], but others disagree with this view [3]. In fact, the shape of the “environmental Kuznets curve” may be different due to the limitations of research indicators and research areas [4]. Although the environmental Kuznets curve still attracts some controversy, these issues do not affect our use of this theory as a logical framework to analyze the relationship between the tourism economy and air pollution. 

The international literature on the relationship between the tourism economy and air pollution comprises multiple different perspective; however, most Chinese scholars tend to agree that air pollution is not conducive to tourism development, and it has a negative impact on tourism’s economic growth [5]. However, in practice, we found some interesting phenomena. The impact of air pollution on tourism’s economic growth may not be significantly negatively correlated. On the one hand, since the reform and opening up, the tourism industry in mainland China has taken off, but the overall air quality has gradually declined, implying that air pollution does not seem to have a significant negative impact on tourism’s economic growth from the time dimension. On the other hand, eastern China is an industrial agglomeration area with relatively serious air pollution, and the tourism industry is also very strong. However, the air quality in western China is much better than that in the eastern region, but the development of the tourism economy is relatively poor. From the spatial dimension, air pollution does not seem to have a significant negative impact on tourism’s economic growth. The emergence of this phenomenon may be the reason why air pollution is only one of many factors that affect the willingness of tourists to travel, and the development of tourism also has a negative effect on the environment. How we re-define the relationship between air pollution and tourism’s economic growth requires in-depth research.

The process of tourism activities involves the pursuit of a pleasant experience. Air pollution will affect tourists’ subjective sensation, which in turn will have a negative impact on the tourism economy. At the same time, the development of the tourism economy is also accompanied by the development of travel, food, accommodation, etc., which will inevitably bring about environmental pollution. The existing literature in China mainly focuses on the impact of the environment on tourism. However, according to the above analysis, the spatio-temporal dynamic relationship between air pollution and the tourism economy is not limited to this, and the reaction of tourism to the environment is also worth studying. In view of this, our paper mainly solves the following problems: First, we want to explorethe correlation between air pollution and the tourism economy in mainland China, and verify the exsitence of “Kuznets” curve; Second, we aim to study whether the tourism economy is included among the driving forces of air pollution, and find out the role the tourism economy plays in it. Third, we would like to put forward some policy suggestions to promote the high-quality development of regional tourism.

## 2. Literature Review

With regard to the relationship between the economy and the environment, Grossman and Krueger (1992) [6] found that when the national income level is low, the concentration of sulfur dioxide and “smoke” increases with the increase in GDP per capita, but when the income level is high, the concentration decreases with the increase in GDP per capita. This phenomenon is similar to the “inverted U-shaped” relationship between income gap and per capita GDP proposed by Kuznets (1955) [7], and so the curve relationship between economic growth and environmental pollution is also known as the “environmental Kuznets curve” (*EKC*). Lopez (1994) [8], Stern (1996) [9], Hartman and Kwon (2005) [10] and other studies also confirmed this view. Some Chinese scholars have found that the environmental Kuznets curve is also applicable in China [11,12]. However, the “inverted U-shaped” curve relationship between the economy and the environment is not absolute. Some scholars believe that the “inverted U-shaped” curve relationship between carbon dioxide emissions intensity and economic development level is only in line with the development of developed countries [13]. There may also be an “inverted N-shaped” curve relationship between economic development and air quality [14]. In addition, urbanization [15], industrial structure [16] and the development of the transportation industry [17] will have a negative impact on air quality.

Similarly, the development of tourism is closely related to air quality. Differing from other industries, while tourism brings pollution, it will also be impacted by pollution. There are some earlier studies on the relationship between air pollution and the tourism economy. For example, air pollution does affect destination choice, and there is a long-term Granger causality between Singapore’s tourism development and carbon emissions [18]. In some developed countries, such as Austria, Belgium, Canada, Chile, Denmark, France, Ireland, Japan, South Korea, Sweden and the United States, the impact of their pollution index (CO_2_ emissions) on international tourism is significantly negative [19]. However, few studies have examined the direct impact of air pollution on tourism, with most of them incorporating it into the category of the impact of climate change on the tourism economy. For example, some scholars believe that atmospheric and climatic conditions will lead to changes in aviation demand for international travel, thereby changing the choice of tourism destinations [20]. Some scholars even believe that air pollution and climate change are nightmares for tourism development [21]. Most of current studies consider the combination of natural and social factors when it comes to analyzing the factors influencing the relationship between air pollution and tourism economy. Taking the Sun Moon Lake scenic spot in Chinese Taiwan as an example, some scholars believe that air pollution and rainfall jointly affect the tourists visiting the scenic region, but they are also affected by the seasonality [22]. 

Tourism development also has a certain adverse effect on the environment [23]; tourism is recognized as an important factor which will result in environmental externalities. The number of tourists increased by 1% and the concentration level of PM_10_ increased by 0.45% [24]. COVID-19 has improved the environment by inhibiting tourism in the Central and Eastern European (CEE) region [25]. To measure the degree of air pollution, the existing literature uses a series of indicators including CO_2_ [19], PM_10_ [24], as well as SO_2_, NO_2_, and O_3_ [26], etc. Some scholars select other relevant indicators such as temperature, precipitation, snowstorms, and sunshine time [27,28], as well as indicators such as the economic development cycle, personal income, and unemployment rate [29,30] for the purpose of exploring the relationship between climate change, macroeconomics and tourism economy from a broader perspective.

Since China has prioritized the development of inbound tourism in recent years, the statistics in this field are relatively complete, making it be possible to study the relationship between inbound tourism and air pollution. Most of the existing literatures in China agrees that air pollution will lead to a decline in inbound tourists, and there is a significantly negative correlation [31]. Some scholars also believe that there is not simply a negative correlation, but rather the impact of air pollution on inbound tourism has a certain lag period [32]. Air pollution is constantly spreading, and air pollution in neighboring provinces has an impact on local inbound tourism, and so spatial factors need to be included in the influence system [33]. With deepening research, the impact of air pollution on inbound tourism has gradually expanded from the economic field to other fields, such as risk perception and tourism experience [34], tourism flow and its spatial spillover [35]. The reverse effect of tourism development on air pollution is also attracting more and more attention. With the continuous development of the economy, China’s support policies for different tourism markets have also changed accordingly. The economic function of inbound tourism has gradually given way to a cultural function. At present, the development policy of China’s tourism industry is “to actively develop inbound tourism, regulative the development of outbound tourism, and comprehensively enhance domestic tourism” [36]. The proportion of domestic tourism in the tourism market is gradually increasing. The relationship and mechanism between the overall tourism economy and air pollution need to be further studied.

In words, our research in this paper has the following conclusions:

(1) From the perspective of index selection, domestic scholars generally choose SO_2_, PM_2.5_, and comprehensive indicators to measure environmental pollution. In recent years, scholars have begun to use the method of questionnaire survey [34].
(2) As for the research methods, spatial-temporal comparison research methods emerge in an endless stream, such as structural equations, bivariate ESDA, impulse response models and so on, which makes the research results more scientific.
(3) Regarding the scope of research, most of the literature focuses on province-level data, although large-scale, space–time comparative research is extant, but is still relatively limited.
(4) Considering the research content, most of the studies tend to study the impact of air pollution on inbound tourism. In fact, the relationship between overall tourism development and air pollution is also particularly important, and the role of tourism on the environment is also worthy of study.
(5) Regarding the research results, most of the domestic studies believe that air pollution has a significant negative impact on the development of tourism economy. In general, the research field of tourism and pollution in China is relatively narrow, ignoring the adverse effects of tourism development, and rarely studying the impact of the tourism economy on air pollution from the more macro perspective of climate change and the regional economy.

The activities of China’s tourism industry show that there is no simple negative correlation between air pollution and the tourism economy. In fact, tourism also reacts to air pollution, and air pollution is not only affected by tourism, but also other social and economic phenomena such as industrial structure, the regional economy, and traffic factors, which also affect the relationship between tourism and pollution. Therefore, on the one hand, the correlation between air pollution and the tourism economy is worthy of further study; on the other hand, this correlation is affected by other social and economic factors. The research on the dynamic relationship between them must be included in the dynamic mechanism of air pollution growth. The research significance of this paper is revealing the temporal relationship between tourism economy and air pollution from the perspective of cities, exploring the role tourism plays in air pollution, and analyzing the factors affecting these roles.

## 3. Data and Research Methods

### 3.1. Data Source and Variable Description

This paper collated the annual panel data of tourism economy, air pollution and other related indicators in various prefecture-level city and municipalities directly under the Central Government (hereinafter collectively referred to as “cities”) in China. The air pollution data were compiled from the atmospheric composition analysis group of Dalhousie University http://fizz.phys.dal.ca/~atmos/martin/?page_id=140 (accessed on 25 January 2022). The wind speed data came from the National Meteorological Science Data Center http://www.cma.gov.cn/2011qxfw/2011qsjgx/ (accessed on 25 January 2022). The rest of the data were obtained from the statistical yearbook of each city unless otherwise specified. Due to the lack of data in some statistical yearbooks in very early or too recent years, the time span of panel data selected in this paper was 2004–2016 for the sake of data integrity. All of the monetary data were adjusted by CPI for each year to remove the effect of price levels. The specific variables are described as follows.

(1) Tourism economic indicators. Here, the annual tourism revenue of each city was selected as the indicator to measure the tourism economy. Tourism revenue was divided into the tourism revenue of domestic tourists, the tourism revenue of inbound tourists, and the sum of the former two, that is, total tourism revenue (unless otherwise specified, the tourism revenue below represents the total tourism revenue). The unit of tourism revenue from inbound tourists was U.S. dollars, which was calculated by converting the average exchange rate (data from the China Foreign Exchange Trade System (http://www.chinamoney.com.cn/chinese/forsddshis/?datatype=3, accessed on 25 January 2022) of U.S. dollars to RMB for each year into RMB. 

(2) Air pollution indicators. Considering the authenticity, authority and sustainability of the data, our paper chose PM_2.5_ concentration data as the measure of air pollution. Specifically, according to the PM_2.5_ concentration data of the Atmospheric Composition Analysis Group of Dalhousie University, the data of each project were matched with the vector map of each city in China to obtain the mean concentration data. The accuracy of the data was high, and the consistency of the statistical caliber was guaranteed, meaning that these data can be used for long-term time series analysis. PM_2.5_ is closely related to various traditional energy consumption and is easily perceived by residents.

In addition to the possible mutual influence of tourism economy and air pollution, air pollution is a factor related to all aspects of social production. The impact of tourism economy on air pollution may be affected by some other economic factors. In order to deeply analyze the impact mechanism, referring to the relevant literature [37], economic, transportation, market, industrial structure and other factors were introduced into the impact measurement system of the tourism economy on air pollution. The specific factors introduced are as follows.

(3) Economic factors (*EC*): expressed by the city’s GDP (billion yuan) in that year. The total GDP can better measure the overall productivity level of a city.

(4) Traffic factors (*RT*): traffic has the ability to guide, support and guarantee regional development, and is an important indicator reflecting the advantages and disadvantages of regional development conditions. In addition, the energy consumption of automobiles is also an important source of PM_2.5_. This paper used the city’s highway mileage to measure the traffic conditions in the region, which can better reflect the road travel conditions of tourists.

(5) Market factors (*MA*): the market is the basis for testing the quality of economic development. This study used the marketization index invented by Fan Gang et al. (2011) to represent market conditions. The score of marketization index is a summary of the scores of five aspects, namely: the relationship between the government and the market, ② the development of the non-state-owned economy, ③ the development degree of the product market, ④ the development degree of the factor market, ⑤ the development of market intermediary organizations and the legal environment. There are several sub indicators under each aspect [38,39]. This index can comprehensively measure the perfection of the market.

(6) Factors of industrial structure: the optimization and rationalization index of industrial structure is a commonly used index in the research of industrial structure [40,41]. This study examined the impact mechanism of air pollution from the perspective of the rationalization of industrial structure (*RIS*) and the optimization of industrial structure (*OIS*). The *RIS* and *OIS* of each city in each year were calculated from the proportion of the output value and employees of the primary, secondary and tertiary industries. Let $h_{1}$, $h_{2}$ and $h_{3}$, respectively, represent the proportion of employees in the first, second and tertiary industries, and $p_{1}$, $p_{2}$ and $p_{3}$, respectively, represent the proportion of output value of the first, second and tertiary industries, and then the specific calculation methods of RIS and OIS are as follows:

$$RIS=h_{1}\ln(\frac{p_{1}}{h_{1}})+h_{2}\ln(\frac{p_{2}}{h_{2}})+h_{3}\ln(\frac{p_{3}}{h_{3}})$$

$$OIS=3\arccos(\frac{h_{1}}{\sqrt{h_{1}^{2}+h_{2}^{2}+h_{3}^{2}}})+2\arccos(\frac{h_{2}}{\sqrt{h_{1}^{2}+h_{2}^{2}+h_{3}^{2}}})+\arccos(\frac{h_{3}}{\sqrt{h_{1}^{2}+h_{2}^{2}+h_{3}^{2}}})$$

(7) Wind speed factor (*WD*): the air flow in an area will also affect the air quality. Here, the annual average wind speed(m/s) of the city was used to measure the air flow in the area.

Due to the acquirement data in the statistical yearbook data, after removing missing values, the data included a total of 13 periods of data from 102 cities across the country. These 102 cities came from 18 provincial administrative units in China and were distributed in various regions, and ranged from the most developed first-tier cities to the least developed fifth-tier cities, and so they had good generality. The variable names and their descriptive statistics are shown in Table 1. It can be seen that the data span of economic factors, air pollution, tourism economy and other indicators was large, indicating that the sample included various types of cities.

### 3.2. Research Method

(1) *PVAR* model. When discussing the impact of the tourism economy on air quality, we need to consider that there is a mutual causality between them. Ignoring the endogeneity caused by mutual causality may confuse causality analysis. The *PVAR* model can accurately reflect the interaction between the two endogenous variables of tourism economy and air pollution, without additional consideration of endogenous treatment [42]. Panel vector autoregression (panel *VAR*, or *PVAR*) builds a model based on the statistical properties of the data, taking each endogenous variable in the system as a function of the lagged values of all endogenous variables in the system to construct the model. Since the structure of the *PVAR* model is not intuitive and concise, the dynamic characteristics of the *PVAR* model can be analyzed by introducing the impulse function. Using impulse response function, we can intuitively observe the response of another variable caused by the change in one variable. Its general form is:

(1) $$M_{t}=f_{j}+A_{1}M_{t-1}+A_{2}M_{t-2}+\ldots +A_{p}M_{t-p}+B_{0}X_{t}+\ldots +B_{r}X_{t-r}+\varepsilon_{t}$$

Among them, $M_{t}$ is the k-dimensional endogenous variable vector, $M_{t-i}$ (*i* = 1, 2, …, *p*) is the lag endogenous variable vector; $X_{t-i}$ (*i* = 0, 1, …, *r*) is the d-dimensional outer variable vector of endogenous variables; and p and r are the lag orders of endogenous and exogenous variables, respectively. $A_{i}$ and $B_{i}$ are parameters to be estimated, where $A_{i}$ is a k × k dimensional coefficient matrix and $B_{i}$ is a k × d dimensional coefficient matrix. The vector $f_{j}$ represents the fixed effects of individual j. $\varepsilon_{t}$ is a vector of k-dimensional random error terms. This study used Stata software to estimate the parameters, and used the impulse response function to further analyze the dynamic impact of the tourism economy on air pollution.

(2) Geodetector. *PVAR* can intuitively show the impact of changes in tourism economy on air quality over a period of time, but it is not conducive to showing the role of the tourism economy in many factors which may affect air quality. In order to show and compare the interpretation degree of tourism economy and other factors on the change in air quality, geographic detectors were used in this paper. Geodetector is a new statistical method to detect spatial heterogeneity and reveal the drivers behind it, without assumptions of linearity; with an elegant form and clear physical meaning, its basic principle is based on the following assumptions: if an independent variable has an important impact on the dependent variable, its distribution should be similar to the dependent variable [43,44]. Its expression is:

(2) $$q=1-\frac{1}{n\sigma^{2}}\sum_{i=1}^{L}n_{i}\sigma_{i}^{2}$$

where q is the interpretation degree of the detection factor to the spatial differentiation of the dependent variable, which takes the value [0, 1]. The larger the value, the greater the influence of the factor on the change in air pollution. Through the comparison of q values among various factors, we can identify the decisive factors affecting the spatial differences of regional air pollution. n is the total number of samples in the study area; $\sigma^{2}$ is the total dispersion variance of the study area; L is the number of samples in the secondary area; and $n_{i}$ and $\sigma_{i}^{2}$ are the sample number and dispersion variance of area i, respectively. The study incorporated the tourism economy into the impact system of air pollution, and selected factors such as the economy, transportation, market, industrial structure and wind for detection and analysis. For details, see the “Indicators and Data” section. This paper used the software Geodetector 1 Software (State Key Laboratory of Resources and Environmental Information System, Beijing, China) http://www.geodetector.cn/ (accessed on 19 January 2022) provided by Wang et al. [43,44].

### 3.3. Variable Calculation and Verification

**Figure 1:**
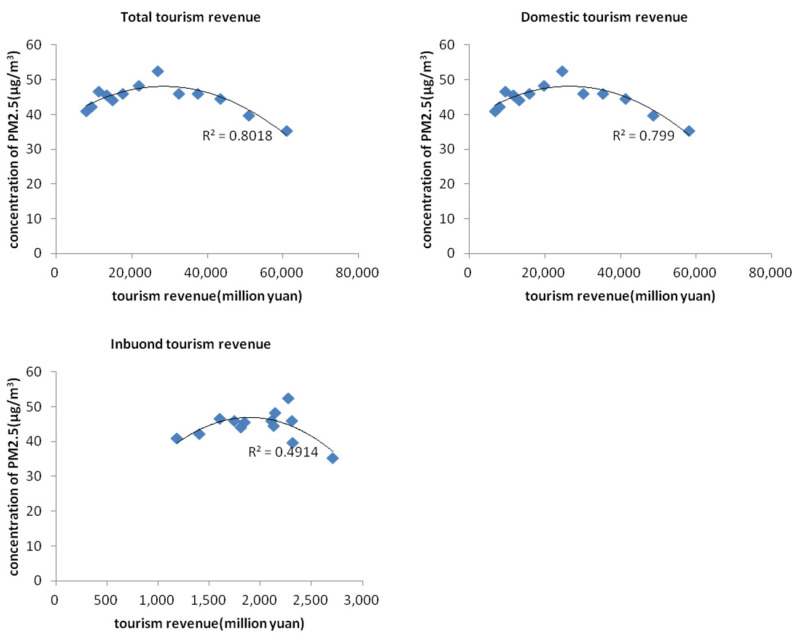
Scatter diagram of total/domestic/inbound tourism revenue and PM_2.5_ concentration.

**Figure 2:**
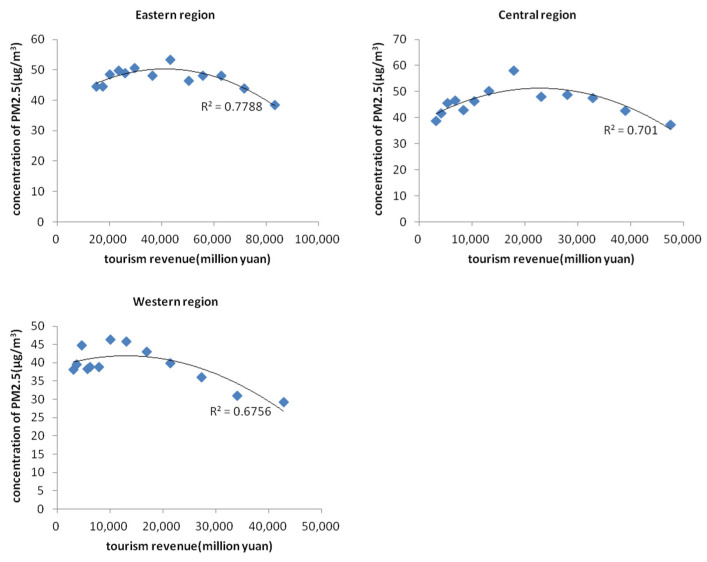
Scatter diagram of tourism revenue and PM_2.5_ concentration in eastern/central/western region.

In order to verify whether the “environmental Kuznets curve” phenomenon in the process of economic development also exists in the tourism development in China, we used the time series data of the average of all cities’ air pollution data and tourism economic data from 2004 to 2016. The scatter plot of tourism income and PM_2.5_ concentration was drawn and fitted with the curve, and the results are shown in Figure 1 and Figure 2. Figure 1 shows the relationship between total tourism revenue, domestic tourist revenue, inbound tourist revenue and PM_2.5_ concentration, respectively. From the curve fitting R^2^, it is shown that there is a certain “inverted U-shaped” relationship between the development of the tourism economy in mainland China and PM_2.5_ emissions, which is generally significant. According to different sources of tourism revenue, the “inverted U-shaped” significance between domestic tourism revenue and PM_2.5_ emission mechanism is stronger than that of inbound tourists (the R^2^ value is larger). Figure 2 shows the scatter plot of the total tourism revenue and PM_2.5_ concentration in the eastern, central and western regions. It can be seen that there is also a certain “inverted U-shaped” relationship between the tourism economic development and PM_2.5_ emissions in the three regions. 

## 4. Empirical Analysis Results

### 4.1. Analysis Results on Time Scale

The natural logarithmic transformation of the data can eliminate the heteroscedasticity and the elastic value between the two, without affecting the cointegration relationship, and can linearize the trend. The data for PM_2.5_ concentration and total tourism revenue, domestic tourism revenue, and inbound tourism revenue panel data were logarithmically processed, and recorded as ln *PM*, ln *TR*, ln *DTR* and ln *ITR*, respectively. First, we tested the stationarity of the data. If the data are non-stationary—that is, if each variable has a certain upward or downward trend over time—they may lead to pseudo regression. Two methods of LLC and IPS were used to test the stationarity of the data. The unit root test of ln *PM*, ln *TR*, ln *DTR* and ln *ITR* did not pass the 10% significance level test, indicating that the data were non-stationary. ln *PM*, ln *TR*, ln *DTR* and ln *ITR* were tested for unit root after first-order difference, respectively, and the results are shown in Table 2. All variables passed the 1% significance level test, indicating that the original data have an integration of order one.

Each variable has the same integration of order, and a cointegration test is required at this time. In this paper, the common cointegration test methods Kao and Pedroni are used to verify whether ln *PM* is cointegrated with ln *TR*, ln *DTR* and ln *ITR*, respectively. The results are shown in Table 3. It is shown that each statistic is significant at the 0.05 level, indicating that ln *TR*, ln *DTR* and ln *ITR* are cointegrated with ln *PM*.

First, we consider the relationship between total tourism revenue and PM_2.5_ concentration in the air. Because both ln *PM* and ln *TR* have an integration of order one and cointegration, the first-order difference is taken. The determination of the lag order is very important for the *PVAR* model. When choosing the lag order, there are usually some contradictions. In order to improve the reflection ability of the dynamic characteristics of the model, the lag order can be increased, but the increase in the lag order will lead to an increase in the number of lag variable coefficients, and the degree of freedom of the model will be decreased. Therefore, when choosing a lag period, both sufficient lag terms and sufficient degrees of freedom should be considered. The Akaike information criterion (*AIC*), Bayesian information criterions (*BIC*), and Hannan-Quinn information criterion (*HQIC*) are usually used as judgment criteria. The results are shown in Table 4. After comprehensive consideration, the optimal lag order is 2.

**Figure 3:**
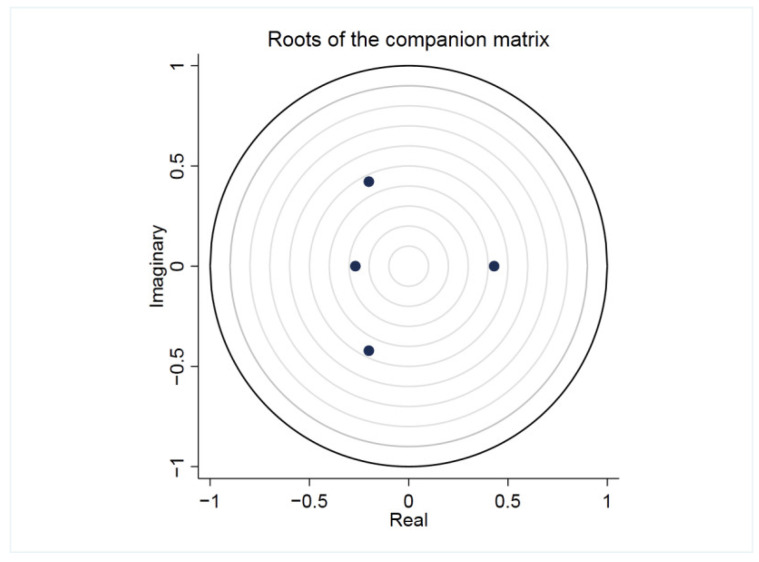
The results of stability test. The largest circle in the figure represents the unit circle.

After the model is established, the stability should be tested to see whether it has economic significance. If the model is unstable, the impulse response function cannot be used to analyze the interaction between variables. We check whether the established *PVAR* model is stable according to the size of the reciprocal modulus of the AR characteristic root. When all of them are less than unit 1, it indicates that the established model is stable. The test results are shown in Figure 3. The modular lengths of the four characteristic roots are all less than 1—that is, the established *PVAR* model is stable.

The Granger causality test is mainly used to describe whether a change in one variable can cause a change in another variable at the level of statistical estimation. If the past value of B can help in the prediction of the A variable, or the correlation between A and B is statistically significant, it can be said that “the A variable is caused by the B variable Granger”. Therefore, this paper conducts the Granger causality test on the sequence to analyze the causal relationship between variables. The results are shown in Table 5, and it is shown that there is Granger causality between Δln *TR* and Δln *PM*.

**Figure 4:**
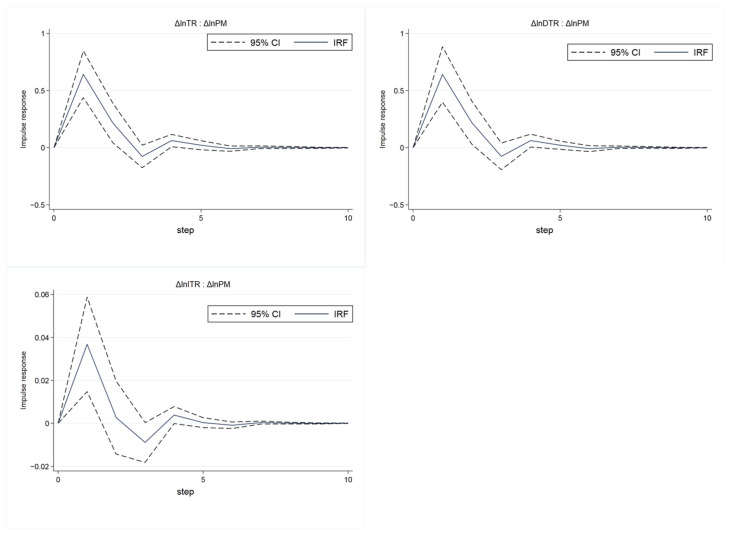
The results of impulse response with total/domestic/inbound tourism revenue as variable.

On the basis that PVAR satisfies stationarity, stability, and Granger causality, the dynamic impact of tourism economy on air pollution can be further analyzed by using the impulse response function (*IRF*). The results are shown in Figure 4, showing the impact of a one-standard deviation shock of Δln *TR* on Δln *PM*, and the solid line reflects the response of Δln *PM* to the change in Δln *TR*. The dotted line represents the 95% confidence interval obtained by 300 operations of the Monte Carlo method. It can be seen from Figure 3 that the impulse response function value is positive in the short term, and the 95% confidence interval is also positive. The positive function value indicates that the change in the tourism economy will cause the air pollution to change in the same direction in the short term. Then, the value of the impulse response function gradually declines, and approaches 0 when it lags behind three phases, and then it is basically stable. In other words, the tourism economy has the greatest positive impact on air pollution in the first period, and then gradually declines. After the third period, its impact is close to 0 and tends to be stable.

To explore the heterogeneity of the impact of domestic tourism revenue and inbound tourism revenue on air pollution, two *PVAR* models are constructed with domestic tourism income and inbound tourism income as variables, respectively. The impulse response function results of the two *PVAR* models are shown in Figure 4, showing the impact of one standard deviation change in domestic/inbound tourism income on air pollution, respectively. It is shown that the results are generally consistent with the impact of total tourism revenue on air pollution.

**Figure 5:**
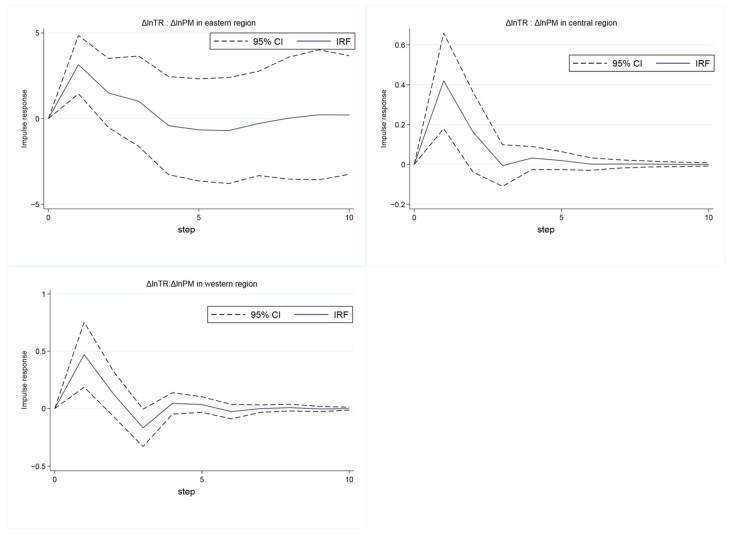
The results of impulse response in east/central/west region.

To explore the heterogeneity of the impact of tourism economy on air pollution in different regions, with reference to the division standard of the Bureau of Statistics, according to the geographical location, policy tendency and economic level, the cities are divided into eastern, central and western regions by region (the eastern region includes 12 provincial administrative units in Beijing, Tianjin, Hebei, Liaoning, Shanghai, Jiangsu, Zhejiang, Fujian, Shandong, Guangdong, Guangxi and Hainan; the central region includes nine provincial administrative units in Shanxi, Inner Mongolia, Jilin, Heilongjiang, Anhui, Jiangxi, Henan, Hubei and Hunan; the western region includes 10 provincial administrative units in Chongqing, Sichuan, Guizhou, Yunnan, Tibet, Shaanxi, Gansu, Ningxia, Qinghai and Xinjiang), and the *PVAR* model is constructed with the total tourism revenue as a variable. The results of the impulse response function are shown in Figure 5, which shows the effect of one standard deviation change in total tourism receipts on air pollution in three regions. The tourism economy in the eastern region has a greater impact on air pollution (the peak of the solid line is about 3, while in other regions the peak is less than 1), and the impact lasts for a longer period of time. This may be because the overall economy in the eastern region is more developed, tourism drives more tourism-affiliated industries and causes pollution, and people from economically developed regions are more likely to travel in private cars that are convenient and fast but consume more energy and pollute more. At the same time, the volatility of shocks in the eastern region is also greater, which may be due to the uneven development of first-tier cities and second- and third-tier cities in the eastern region.

In order to preliminarily verify that the characteristics of the impulse response function in the eastern region are because the overall level of economic development in the eastern region is higher and more unbalanced, Table 6 shows the mean and standard deviation of GDP in the eastern, central and western regions. It can be seen that the average and standard deviation of GDP in the eastern region are significantly higher than those in other regions.

### 4.2. Exploration of Factors Influencing the Air Pollution

The development of air pollution is always constrained by many factors in the national economy. Based on the relevant literature [37], factors such as the economy, transportation, market, and industrial structure were selected, while tourism development was also included as one of influencing factors of air pollution, and then Geodetector was used to analyze the effects of factors and their interactions on air pollution. In order to examine the heterogeneity of the impact of domestic tourism and inbound tourism on air pollution, we divided the tourism economy into two parts, domestic tourism revenue and inbound tourism revenue.

The results are shown in Table 7. We can find that, as a single factor, except for the western region, *RIS* has the greatest impact on air pollution, indicating that in China, the industrial structure is the core factor affecting air pollution. The GDP of the western region is the core factor affecting air pollution. The influence of the dominant interaction factor on air pollution exceeds that of a single factor, indicating that air pollution is more a result of a “joint force”. *RIS* is included in the dominant interaction factor in every region, which further illustrates the importance of industrial structure to air pollution. Tourism is only one of the factors affecting air pollution at the current stage. In the eastern region, the single effect of air pollution on tourism economic growth is relatively large, which is generally consistent with the above analysis. Additionally, inbound tourism has a greater impact on air pollution than domestic tourism.

**Figure 6:**
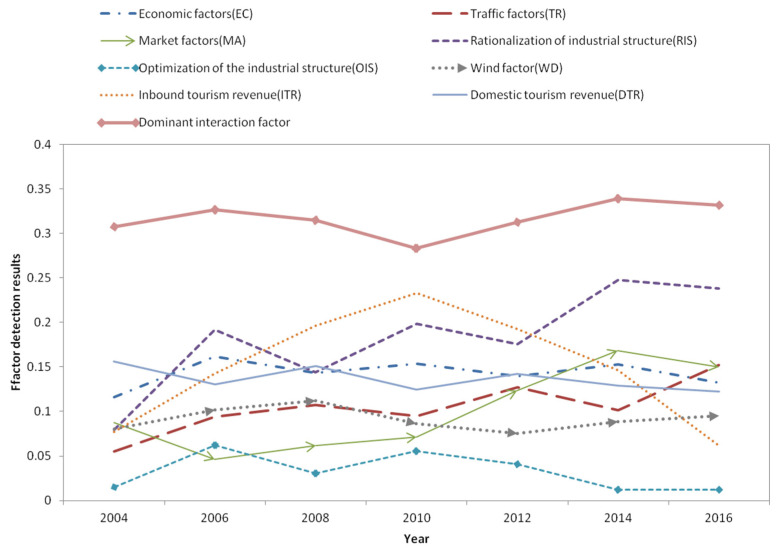
Exploration results of influencing factors of air pollution in main years.

The overall impact of various factors on the tourism economy is analyzed above. Whether it is robust in each year still needs to be verified, so we use cross-sectional data to perform Geodetector analysis. As Figure 6 shows, the tourism economy of each region is one of the many factors affecting air pollution. The impact of inbound tourism on air pollution is relatively high, and has a trend of first rising and then falling, while the impact of domestic tourism on air pollution is generally unchanged. This may be the result of the joint action of economic and social development and the improvement of social environmental protection awareness. The influence of other single factors on the tourism economy is different. The impact of traffic factors, marketization factors, and industrial structure rationalization factors on air pollution is generally on the rise. The impact of economic factors on air pollution remains broadly unchanged. This shows that the impact of marketization and industrial structure on air pollution is becoming more and more critical. The rise in the influence of traffic factors may be the result of the increasing popularity of private cars. It can also be seen from Figure 4 that the overall impact of the interacting leading factors on the tourism economy is still greater than the impact of a single factor on the tourism economy, indicating that the “joint force” factor is still the dominant factor in the development of the regional tourism economy.

Geographic detectors can detect the influence of each factor and its dominant interaction factors in the air pollution impact system. The results of geographic detector analysis show that the factors affecting air pollution also have spatial and temporal scale effects. Under different time and space conditions, the effects of various factors on air pollution are different. Air pollution is affected by many factors, and tourism is just one of them. A single core factor has an important impact on air development, but the dominant interaction factor is the core driving force for the regional air pollution.

Considering the availability of data, the data in this paper are from 2016. According to the curve trend in Figure 6, it can be inferred that during the period from 2017 to now, industrial structure and market factors may be the factors that have had the greatest impact on air quality. The impact of domestic tourism revenue on air quality maintains a medium level, while inbound tourism revenue has less impact on air quality.

## 5. Conclusions

Using the inter-city air pollution and related data in mainland China from 2004 to 2016, this paper analyzes the relationship between air pollution and tourism growth, and its impact mechanism. The results show that there is an indeed an “inverted U-shaped” relationship between tourism economic development and PM_2.5_ emissions, and the tourism economy has a positive impact on air quality in the short term. This impact is generally consistent between domestic tourism revenue and inbound tourism revenue, but there is heterogeneity among different regions, which is manifested in the greater magnitude and volatility of the impact in the eastern region. The heterogeneity among regions may be due to insufficient and uneven economic and social development in mainland China. The results of Geodetector analysis show that the factors affecting air pollution have spatial and temporal scale effects, and the effects of each factor on air pollution are slightly different in different regions and different years. In general, the influence of dominant interaction factors on air pollution is greater than that of a single factor, and the tourism economy is only one factor which affects the air pollution. This research is almost in line with the current reality of tourism development in China, and can provide theoretical guidance for the sustainable development of regional tourism.

Hence, the high-quality development of regional tourism should pay attention to low-carbon tourism and green travel. We need to pay more attention to the “joint force” impact of the tourism economy and other socio-economic factors on air pollution. Therefore, the development of the tourism industry requires “thinking outside of the box”. The development of the tourism industry should not be limited to the economic benefits brought by tourism. The concept of “green” tourism should be the primary task of high-quality tourism development. The implementation of “green” tourism development requires systematic attention to the “joint forces” in the system. Reducing the air pollution caused by tourism is one of an important means to promote “green” tourism, but it must be closely aligned with macroeconomic development, the market system, the industrial structure, and infrastructure improvement.

## Figures and Tables

**Table 1:** Variable names and descriptive statistics.

Variable	Introduction	Obs	Mean	Std. Dev.	Min	Max
Air pollution (*PM*)	Concentration of PM2.5 (μg/m^3^)	1326	44.45	18.15	3.13	108.94
Economic factors (*EC*)	GDP of cities (billion yuan)	1326	209.11	311.26	5.04	2817.87
Traffic factors (*TF*)	Highway mileage of cities (km)	1326	10,977.85	5560.94	1053.00	27,601.00
Market factors (*MA*)	An index for evaluating Marketability progress	1326	9.19	2.53	2.53	16.95
Rationalization of industrial structure (*RIS*)	An index for evaluating the rationalization of industrial structure	1326	0.26	0.21	−0.03	1.05
Optimization of the industrial structure (*OIS*)	An index for evaluating the optimization of industrial structure	1326	6.44	0.38	5.43	7.60
wind factor (*WD*)	Annual average wind speed (m/s)	1326	2.06	0.47	0.97	3.42
Inbound tourism revenue (*ITR*)	Income from inbound tourists (million yuan)	1326	1967.60	5930.33	0.04	43,733.98
Domestic tourism revenue (*DTR*)	Income from domestic tourists (million yuan)	1326	24,885.63	45,565.05	116.80	468,300.00

**Table 2:** The results of stationarity test.

	LLC		IPS	
	Statistic	*p*-Value	Statistic	*p*-Value
ln *PM*	5.351	1.000	0.849	0.802
Δln *PM*	−11.521	0.000	−15.355	0.000
ln *TR*	36.768	1.000	9.110	1.000
Δln *TR*	−18.310	0.000	−18.310	0.000
ln *DTR*	36.710	1.000	9.264	1.000
Δln *DTR*	−18.130	0.000	−10.647	0.000
ln *ITR*	4.788	1.000	11.944	1.000
Δln *ITR*	−17.697	0.000	−13.085	0.000

**Table 3:** The results of cointegration test.

	Kao		Perdroni	
	Statistic	*p*-Value	Statistic	*p*-Value
ln *PM* and ln *TR*	7.704	0.000	10.763	0.000
ln *PM* and ln *TR*	1.733	0.042	6.289	0.000
ln *PM* and ln *TR*	7.091	0.000	10.760	0.000

**Table 4:** The results of optimal lag order selection.

Lag	AIC	BIC	HQIC
1	−1.3538	−0.3544	−0.9738
2	−1.73859 *	−0.392804 *	−1.0773
3	−1.0669	0.17262	−0.5905
4	−1.5008	−0.3363	−1.19627 *

Note: * indicates that the statistics are the smallest, that is, the order is the best.

**Table 5:** The results of the Granger causality test.

Excluded	Dependent Variable: Δln *PM*		Dependent Variable: Δln *TR*	
	Δln *TR*	ALL	Δln *PM*	ALL
Statistic	35.836	35.836	4.942	4.942
*p*-value	0	0	0.085	0.085

**Table 6:** GDP of the eastern, central and western regions.

GDP (Billion Yuan)	Obs.	Mean	Std. Dev.
eastern	546	325.40	420.82
central	390	142.77	170.50
western	390	112.65	141.78

**Table 7:** Exploration results of influencing factors of air pollution.

Detection Factor	Whole Country	Eastern Region	Central Region	Western Region
Economic factors (*EC*)	0.112	0.184	0.228	0.328
Traffic factors (*TF*)	0.067	0.157	0.250	0.093
Market factors (*MA*)	0.075	0.110	0.246	0.226
Rationalization of industrial structure (*RIS*)	0.175	0.219	0.278	0.170
Optimization of the industrial structure (*OIS*)	0.021	0.037	0.258	0.198
Wind factor (*WD*)	0.095	0.076	0.112	0.125
Inbound tourism revenue (*ITR*)	0.153	0.151	0.093	0.083
Domestic tourism revenue (*DTR*)	0.124	0.112	0.090	0.096
The q-value of dominant interaction factor	0.410	0.457	0.419	0.451
Dominant interaction factor	*EC* ∩ *RIS*	*MA* ∩ *RIS*	*TR* ∩ *RIS*	*EC* ∩ *RIS*

## Data Availability

The data that support the findings of this study are openly available in National Bureau of Statistics of China.
